# Phenylephrine Affects Peripapillary Retinal Vasculature—an Optic Coherence Tomography Angiography Study

**DOI:** 10.3389/fphys.2017.00996

**Published:** 2017-12-04

**Authors:** Jingyi Cheng, Jian Yu, Chunhui Jiang, Xinghuai Sun

**Affiliations:** ^1^Department of Ophthalmology and Visual Science, Eye, Ear, Nose and Throat Hospital, Fudan University, Shanghai, China; ^2^Key Laboratory of Myopia, Ministry of Health, Fudan University, Shanghai, China; ^3^Shanghai Key Laboratory of Visual Impairment and Restoration, Fudan University, Shanghai, China

**Keywords:** mydriatic eye drops, phenylephrine, tropicamide, retinal vessels, peripapillary retinal vasculature, optic coherence tomography angiography, healthy volunteers

## Abstract

**Purpose:** To evaluate and compare the effect of topical mydriatic eye drops (0.5% tropicamide alone, and a 0.5% tropicamide/0.5% phenylephrine mixture) on the retinal perfusion using optic coherence tomography angiography (OCTA).

**Methods:** The perfused vessel density in the macular and peripapillary areas of both eyes was measured in eight healthy subjects using OCTA and a split-spectrum amplitude decorrelation angiography algorithm (SSADA). Various parameters, including retinal vessel density, were obtained at baseline and again 30 min after the instillation of either the tropicamide or the tropicamide/phenylephrine mixture eye drops in the right eye.

**Results:** Following instillation of the tropicamide/phenylephrine mixture eye drops, there was a significant reduction of vessel density in the peripapillary area (from 89.63 ± 4.53% to 85.00 ± 4.14%, *P* = 0.034), but not in the macular areas. The mean effect size in the peripapillary area was −4.63%, and was not correlated with age, sex, or baseline vessel density. There was no reduction in vessel density *i*n the macular or peripapillary areas after instillation of 0.5% tropicamide alone, and no reduction in vessel density in the contralateral (control) eyes.

**Conclusion:** Topical phenylephrine reduced the retinal vessel density within the peripapillary area, but not within the macular area. Therefore, studies using OCTA, especially those focusing on the peripapillary area, should take into account this effect of phenylephrine on vessel density.

## Introduction

Mydriatic agents, such as the antimuscarinic tropicamide or the α-adrenergic agonist phenylephrine, are used for pupil dilation prior to intraocular surgery and fundal examination (Saint-Martin, 1929; Heath, 1936; Haddad et al., 1970; Duffin et al., 1983; Gimbel, 1989). A tropicamide/phenylephrine mixture achieves greater maximum pupil dilation than each individual agent at an equivalent concentration (Fraunfelder, 1999; Park et al., 2009), and is therefore commonly used (Duffin et al., 1983; Fraunfelder, 1999; Takayama et al., 2004, 2009; Mayama et al., 2010). Phenylephrine is an α1-adrenergic receptor agonist that induces vasoconstrictive activity in the peripheral vessels, including the conjunctival vessels (Heath, 1936) and anterior ciliary arteries (Van Buskirk et al., 1990). It has been shown that the topical instillation of phenylephrine can lead to a reduction of blood velocity in the optic nerve head (ONH) in rabbits, monkeys, and healthy young human subjects (Takayama et al., 2003, 2004, 2009; Mayama et al., 2010; Vandewalle et al., 2013). However, due in part to the measuring techniques employed, most previous studies have focused on its effect on the large vessels of the ONH rather than on the vessels in other parts of the retina, and these have not been fully investigated. We have recently reported a large and significant difference in the response to hypoxia between retinal vessels in the macular and the peripapillary areas (Xu et al., 2016). Accordingly, it would be interesting to investigate the effects of topical mydriatic eyedrops on retinal vessels in different areas of the fundus.

Optic coherence tomography angiography (OCTA) is a new imaging technique that enables evaluation of the retinal vasculature to the capillary level (Jia et al., 2012a) and has been employed in research into ocular diseases such as glaucoma, diabetic retinopathy, age-related macular degeneration, and artery and vein occlusions (Carlo et al., 2015). To improve the image quality of this technique, eye drops of a tropicamide/phenylephrine mixture is commonly used to dilate the pupil. Therefore, it is essential to investigate the effects of topical mydriatic eye drops on the retinal vessels.

We hypothesized that phenylephrine would reduce retinal perfusion in all assessment regions; in this study, we used OCTA to evaluate the effects of commonly used topical mydriatic agents, especially phenylephrine, on the retinal vasculature in the peripapillary and parafoveal areas.

## Methods

### Study subjects

This study was performed in accordance with the tenets of the Declaration of Helsinki and was approved by the Medical Ethics Committee of the Eye and ENT Hospital, Fudan University, Shanghai, China (approval #2014043). Written informed consent was obtained from all subjects after the study had been explained. Healthy and non-smoking volunteer subjects were recruited between April and June, 2015. The subjects underwent a complete ophthalmic examination including medical history, best corrected visual acuity (BCVA; E-charts at a distance of 5 m), slit-lamp biomicroscopy (YZ5E, 66 Vision Tech. Co., Suzhou, China), non-contact intraocular pressure (IOP) tonometry (Full Auto Tonometer TX-F, Canon, Japan), and axial length (AL) measurement (IOLMaster 500, Zeiss, Germany).

Subjects were included only if both eyes met the following inclusion criteria: (1) BCVA > 0.8; (2) refractive error between +1 and −6 diopters; (3) IOP ≤ 21 mmHg; (4) AL of 20–26 mm. The exclusion criteria were the presence of any ocular disease or the use of any medication that could affect the circulation.

### Pupil dilation test

Two mydriatic methods were used: tropicamide eye drops (1 ml, tropicamide 5 mg; Wuxi Shanhe Group Co., Ltd.) and eye drops containing a mixture of 0.5% tropicamide/0.5% phenylephrine (1 ml, tropicamide 5 mg + phenylephrine 5 mg; Santen Oy). The eye drops were instilled only in the right eyes of the subjects, and the left eyes were used as the controls. The pupils were dilated with the tropicamide eye drops at the first visit and the tropicamide/phenylephrine mixture eye drops at the second visit, after an interval of at least 1 week.

At each visit, baseline OCTA imaging, heart rate (HR), blood pressure (BP), and IOP were recorded in each subject. The mydriatic eye drops were then applied to the right eye every 10 min, for a total of three instillations. Ten minutes after the last drop (i.e., 30 min after the first drop, which is the typical time reported for clinical ophthalmological assessment) (Manny et al., 1993; Park et al., 2009; Tsui et al., 2013), HR, BP, and IOP were recorded and OCTA imaging was performed again. The mean arterial pressure (MAP) was calculated as the diastolic blood pressure (DBP) plus one-third of the difference between the diastolic blood pressure and the systolic blood pressure (SBP). The ocular perfusion pressure (OPP) was determined by subtracting IOP from MAP. An investigator, blinded to the type of eye drop used, recorded all data.

### OCT data acquisition and processing

The principle of OCTA and SSADA has been previously described by Jia et al. (2012b). In brief, speckle variance and decorrelation can detect optical scattering from moving particles such as red blood cells. The contrast between the decorrelation of blood flow and static tissue is used to extract flow signals and produce an angiographic image. In the present study, OCTA scans were obtained with a spectral-domain system (software version 2.0.5.39; Optovue, Inc., Fremont, CA, USA) having an A-scan rate of 70,000 scans per second, a light source centered at a wavelength of 840 nm, and a bandwidth of 45 nm. After the head was stabilized with chin and forehead rests, the subject was asked to fixate on a flashing internal target light. Both eyes were assessed simultaneously. Three dimensional (3D) OCTA scans were acquired over 4.5 × 4.5 mm around the optic disc and over 6 × 6 mm in the macular region, using two repeated B-scans at 304 raster positions, each B-scan consisting of 304 A-scans. Two volumetric raster scans, a horizontal-priority (x-fast) scan and a vertical-priority (y-fast) scan, were obtained consecutively and motion artifact was removed by 3D orthogonal registration and merging of the two scans. An enface retinal angiogram was created by projecting the flow signal internal to the retinal pigment epithelium. All processing was performed using the provided RTVue-XR Avanti software (version 2.0.5.39).

The primary endpoint was altered systemic and ocular parameters, especially the vessel density in the peripapillary, perifoveal and parafoveal areas at 30 min after the instillation of the tropicamide or tropicamide/phenylephrine mixture eye drops.

The peripapillary area was defined as a 700 mm wide elliptical annulus extending outward from the optic disc boundary. The parafoveal area was defined as an annulus centered at the fovea with an outer diameter of 3 mm and an inner diameter of 1 mm, and the perifoveal area was defined as an annulus centered at the fovea with an outer diameter of 5 mm and an inner diameter of 3 mm. The foveal avascular zone (FAZ) was outlined and measured in images that had been magnified six times (using ImageJ software 1.6.0), as described in previous studies (Yu et al., 2015). Vessel density was defined as the percentage of the area occupied by vessels within the segmented area, as acquired by the provided RTVue-XR Avanti software (version 2.0.5.39).

Effect size was defined as the change in vessel density after the instillation of tropicamide or tropicamide/phenylephrine mixture eye drops, and was calculated as post-dilation vessel density minus pre-dilation vessel density. OCTA acquisition and processing were performed by an operator blinded to the type of eye drop used.

### Statistical analysis

The statistical analyses were performed using a commercially available statistical software package (SPSS for Mac, version 22.0). Continuous variables were expressed as the means and standard deviations (SDs). Wilcoxon matched-pairs signed-rank test was used to compare measurements obtained before and after the application of the eye drops. Statistical significance was defined as *P* < 0.05.

## Results

A total of 16 eyes of 8 eligible participants were included in this study (4 males, 4 females; mean age, 30.13 ± 4.12 years, age range, 24–36 years). There was no significant difference in AL or refractive error between the right and left eyes (*P* > 0.05).

### Effect of mydriatic eye drops

At 30 min after the initial administration of the tropicamide or tropicamide/phenylephrine mixture eye drops, there was no change in the values of SBP, DBP, HR, MAP, IOP, and OPP in the right or left eyes (Supplementary Table 1).

**Figure 1:**
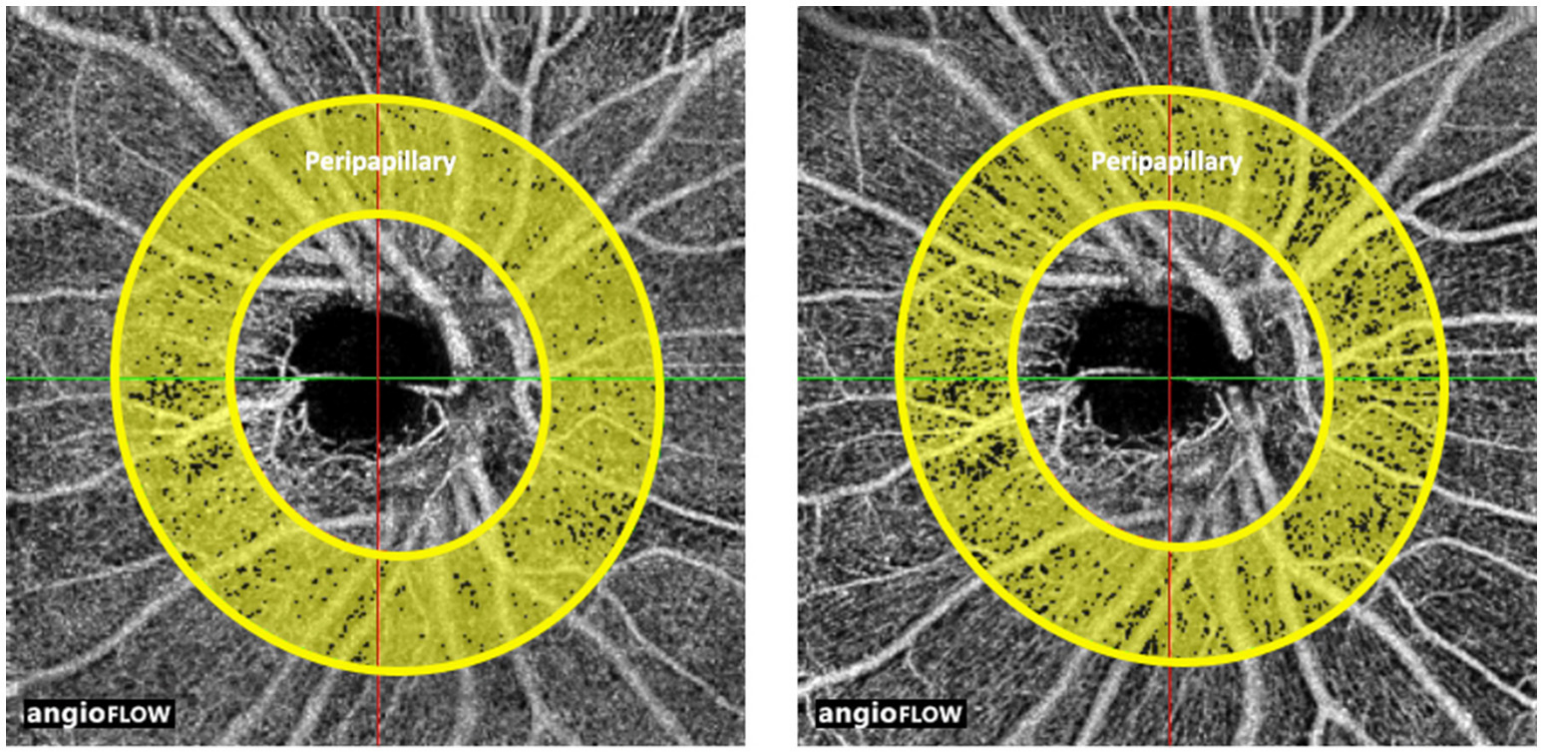
Optic coherence tomographic angiogram of the peripapillary area from a 29 year-old male; showing the annulus between the inner and outer circles, before (left, vessel density 90%) and after (right, vessel density 78%) the instillation of tropicamide/phenylephrine mixture eye drops. The yellow shading represents the perfused vessel density.

**Figure 2:**
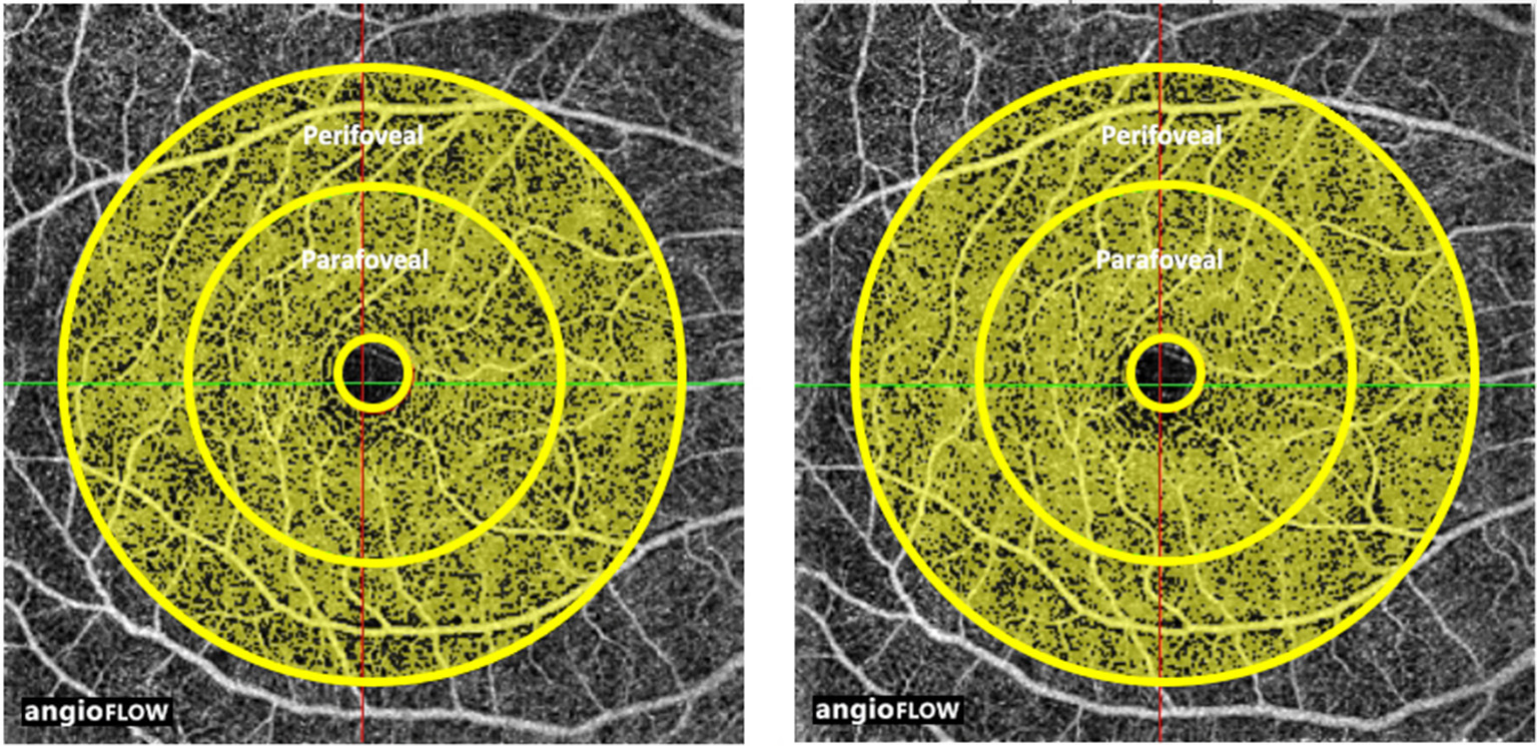
Optic coherence tomographic angiogram of the macular area from a 29 year-old male; showing the perifoveal area, the annulus between the middle and outer circles, before (left, vessel density 79%) and after (right, vessel density 73%), and the parafoveal area, the annulus between the inner and middle circles, before (left, vessel density 81%) and after (right, vessel density 76%) the instillation of tropicamide/phenylephrine mixture eye drops. The yellow shading represents the perfused vessel density.

**Figure 3:**
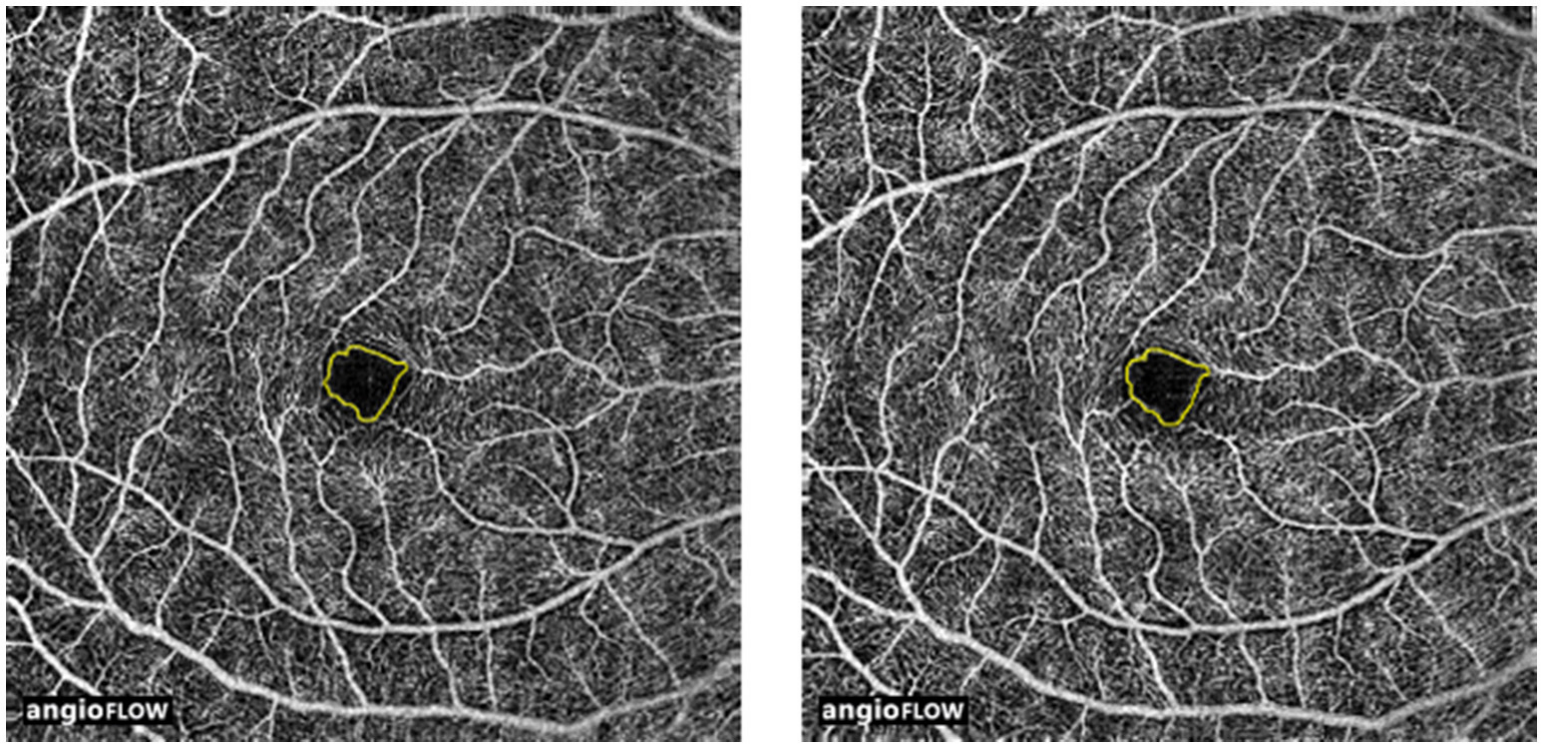
Optic coherence tomographic angiogram of the foveal avascular zone (FAZ) from a 29 year-old male; showing outlined and measured FAZ before (left, 0.38 mm^2^) and after (right, 0.38 mm^2^) instillation of tropicamide/phenylephrine mixture eye drops.

In the right eyes treated with tropicamide/phenylephrine mixture (Individual data were shown in Supplementary Table 2), the vessel density in the peripapillary area was significantly reduced after instillation of the eye drops (from 89.63 ± 4.5 3% to 85.00 ± 4.14%, *P* = 0.034) (Table 1, Figure 1). The effect size in the peripapillary area was −4.63 ± 4.63%, and was not related to age, sex, or the baseline retinal vessel density. And there was little change of vessel density in the peripapillary area after instillation of tropicamide in the right eyes (Table 1, Supplementary Figure 1). There was no change in vessel density in the perifoveal, parafoveal areas, and FAZ after the instillation of the tropicamide or the tropicamide/phenylephrine mixture eye drops (Table 1, Figures 2, 3 and Supplementary Figures 2, 3). In the control eyes (left, which received no eye drops), there was no change in vessel density in the peripapillary or macular areas after application of the two types of eyedrops to the treated right eyes (Table 1).

**Table 1:** Retinal vessel density before and after instillation of tropicamide or tropicamide/phenylephrine mixture eye drops.

		**Tropicamide**				**Tropicamide/Phenylephrine mixture**			
**Area**	**Eyes**	**Pre-dilation**	**Post-dilation**	**Change**	*P*-value^*^	**Pre-dilation**	**Post-dilation**	**Change**	*P-*value^*^
Peripapillary	OD (Tested eyes)	90.38 ± 3.02%	90.13 ± 4.05%	−0.25 ± 2.87%	0.916	89.63 ± 4.53%	85.00 ± 4.14%	−4.63 ± 4.63%	0.034^*^
	OS (Control eyes)	88.88 ± 3.00%	89.63 ± 3.70%	0.75 ± 3.45%	0.552	88.75 ± 2.49%	89.75 ± 3.37%	1.00 ± 2.83%	0.478
Perifovea	OD (Tested eyes)	76.88 ± 2.64%	77.38 ± 3.07%	−0.50 ± 1.77%	0.391	76.63 ± 5.48%	75.00 ± 5.24%	−1.88 ± 5.64%	0.397
	OS (Control eyes)	71.38 ± 5.34%	72.13 ± 7.70%	0.75 ± 3.45	0.888	71.50 ± 5.42%	73.00 ± 3.96%	1.50 ± 2.83	0.181
Parafovea	OD (Tested eyes)	79.75 ± 2.19%	78.63 ± 3.25%	−1.13 ± 2.64%	0.234	78.38 ± 5.26%	77.50 ± 4.31%	−0.88 ± 5.22%	0.528
	OS (Control eyes)	73.25 ± 4.77%	74.38 ± 6.78%	1.13 ± 5.33%	0.673	73.13 ± 6.10%	74.75 ± 4.03%	1.63 ± 3.54%	0.200
FAZ (mm^2^)	OD (Tested eyes)	0.32 ± 0.08	0.32 ± 0.08	0.00 ± 0.02	0.915	0.32 ± 0.08	0.32 ± 0.08	0.00 ± 0.02	0.748
	OS (Control eyes)	0.35 ± 0.09	0.35 ± 0.09	0.00 ± 0.02	0.739	0.34 ± 0.08	0.35 ± 0.08	0.01 ± 0.02	0.086

*Continuous variables are given as means ± SD. Wilcoxon matched-pairs signed-rank test was used to compare values obtained before and after administration of tropicamide or tropicamide/phenylephrine eye drops. OD, right eye; OS, left eye; FAZ, foveal avascular zone*.

* *P < 0.05; Wilcoxon matched-pairs signed-rank test before and after administration of tropicamide or tropicamide/phenylephrine mixture eye drop*.

## Discussion

In this study, we used OCTA to compare for the first time the effect of topical 0.5% tropicamide and 0.5% tropicamide/0.5% phenylephrine mixture eye drops on vessel density in different parts of the fundus. The tropicamide/phenylephrine mixture eye drops significantly reduced the retinal vessel density in the peripapillary area, while tropicamide eye drops had no effect on vessel density in this area. This finding indicates that the effect on the retinal vessels was attributable to the direct effect of topical phenylephrine. Although there was a significant reduction in vessel density within the peripapillary areas of the treated eyes, vessel density within the macular areas remained unchanged, for reasons that are not fully understood. As expected, we found no change in SBP, DBP, HR, MAP, IOP, and OPP in either eye, and no change in the vessel density of the left (control) eye, confirming that phenylephrine exerts localized, rather than systemic effect. Mizuno et al. (2002) found that at 15 min after topical instillation of nipradilol eye drops, the drug reached a relatively high concentration in the posterior periocular tissue and the posterior retina–choroid and concluded that topically instilled drugs can penetrate from the conjunctival cul-de-sac, through the periocular tissue, to reach the retrobulbar region, where the drug accumulates to a pharmacologically active level around the short posterior ciliary arteries (SPCAs) and central retinal artery (CRA). In the retrobulbar space, the CRA enters the optic nerve ~10 mm behind the eye, whereas the SPCAs wind uncovered across the retrobulbar space (Cioffi et al., 2003). Therefore, the SPCAs should be more affected by phenylephrine than the CRA. The vessels in the macular area originate solely from the CRA, whereas some small vessels in the peripapillary area receive blood flow from the SPCAs, suggesting that our results could be due to this difference in vessel supply. Furthermore, after topical instillation, the drug could also pass into the eye (Mizuno et al., 2002; Del-Amo and Urtti, 2008) and affect the retinal vessels directly. The microvessels in the macular area lack smooth muscle and are largely controlled by pericytes, which are responsive only to α2 adrenergic receptors (Ferrari-Dileo et al., 1992; Rosa et al., 2006); in contrast, the relatively large vessels in the peripapillary area have α1 adrenergic receptors (Ferrari-Dileo et al., 1990). Because phenylephrine is an α1 adrenergic agonist, this difference could also partly explain why significant change was recorded only in the peripapillary area.

Our observations may explain a response to stress-induced catecholamine release (Zacny et al., 1994). A previous study revealed that under stress, subjects exhibited a narrowing of the peripheral visual field while central visual acuity performance remained high (Hammond, 2000). This is in accordance with the present finding that the vessels of the macula, which provides the central vision, remained unchanged; whereas there was a decrease in vessel density at the peripapillary area, which includes vessels supplying the retina that providing the peripheral visual field. The fact that the vessel density of the macula does not change in response to phenylephrine might be a result of natural evolution to enable humans to survive a crisis. Further studies are needed to gain a deep insight into this phenomenon.

Here we employed OCTA, which has been used in previous studies that evaluated the retinal and choroidal vasculature down to the capillary level. Compared with fluorescein angiography and indocyanine green angiography as the gold standards of retinal angiography, OCTA is non-invasive, it uses motion contrast instead of intravenous dye, can be obtained within seconds, and can provide accurate size and localization information with high intravisit repeatability and reproducibility. Although currently limited by its small field of view, continuous development of the technique and software could enable OCTA to become a useful tool in future research into the retinal vasculature.

Takayama et al. reported a 4.4% reduction in the blood velocity of the ONH following topical phenylephrine (Takayama et al., 2003), similar to the 4.63% decrease in vessel density in the peripapillary area observed in the present study. This ~4% decrease was comparable or even larger than the 1.8% reduction in microvessels found in high myopia patients by Yang et al. (2016), and the 2% decrease in anterior diameter in patients with uncontrolled hypertension found by Wong et al. (2003). Therefore, the reduction of vessel density in the peripapillary area caused by topical phenylephrine should be borne in mind when phenylephrine may be unfavorably used in patients that already have impairment of the retinal vasculature, especially those with severe damage of the optic disc vessel. In addition, OCTA is increasingly being used for clinical research into ocular diseases, especially those affecting the retinal vascular system, and mydriasis is often a necessary step to obtain a high-quality image (Tsui et al., 2013). Our results suggest that future studies should take the effect of phenylephrine into consideration.

The present study was limited by the small number of subjects, and by the fact that only young and middle-aged healthy Chinese subjects were included. Therefore, it is necessary to investigate the effects of mydriatic agents on retinal vessel density in the elderly and in symptomatic patients, who are the most likely to require mydriatic agents prior to fundus examinations. Study including a larger number of subjects especially elder ones would improve our knowledge in this field.

In conclusion, this OCTA study revealed that topical phenylephrine, a widely used adrenergic agonist that elicits vascular bed constriction, stimulates a reduction in vessel perfusion in the peripapillary area but not in the parafoveal area.

## Author contributions

JC: contributions to the conception or design of the work; do the acquisition, analysis, or interpretation of data for the work; write the work; final approval of the version to be published; agreement to be accountable for all aspects of the work in ensuring that questions related to the accuracy or integrity of any part of the work are appropriately investigated and resolved. JY: discuss the design of the work; do the acquisition of data for the work; draft the work. CJ: contributions to the conception or design of the work; do the analysis and interpretation of data for the work; revise the work critically for important intellectual content; final approval of the version to be published; agreement to be accountable for all aspects of the work in ensuring that questions related to the accuracy or integrity of any part of the work are appropriately investigated and resolved. XS: have the conception of the work; do the interpretation of data for the work; revise the work critically for important intellectual content.

### Conflict of interest statement

The authors declare that the research was conducted in the absence of any commercial or financial relationships that could be construed as a potential conflict of interest.

## Supplementary material

Supplementary Figure 1 Optic coherence tomographic angiogram of the peripapillary area from a 29 year-old male; showing the annulus between the inner and outer circles, before (left, vessel density 90%) and after (right, vessel density 91%) the instillation of tropicamide eye drops. The yellow shading represents the perfused vessel density.

Supplementary Figure 2 Optic coherence tomographic angiogram of the macular area from a 29 year-old male; showing the perifoveal area, the annulus between the middle and outer circles, before (left, vessel density 71%) and after (right, vessel density 71%), and the parafoveal area, the annulus between the inner and middle circles, before (left, vessel density 80%) and after (right, vessel density 79%) the instillation of tropicamide eye drops. The yellow shading represents the perfused vessel density.

Supplementary Figure 3 Optic coherence tomographic angiogram of the foveal avascular zone (FAZ) from a 29 year-old male; showing outlined and measured FAZ before (left, 0.36 mm^2^) and after (right, 0.37 mm^2^) instillation of tropicamide/phenylephrine mixture eye drops.
