# Supplementary figures and images for: Phenylephrine Affects Peripapillary Retinal Vasculature—an Optic Coherence Tomography Angiography Study
Source: Front Physiol. 2017 Dec 4;8:996. doi: 10.3389/fphys.2017.00996 (PMC5722831; doi:10.3389/fphys.2017.00996)

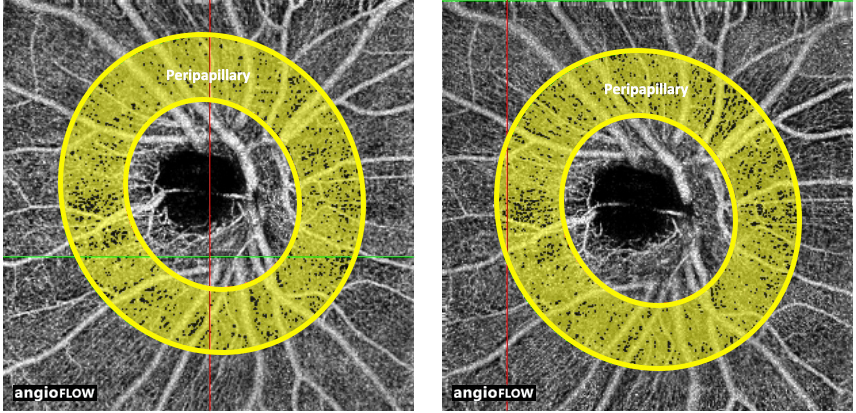

Supplement: Supplementary Figure 1 — Optic coherence tomographic angiogram of the peripapillary area from a 29 year-old male; showing the annulus between the inner and outer circles, before (left, vessel density 90%) and after (right, vessel density 91%) the instillation of tropicamide eye drops. The yellow shading represents the perfused vessel density. [file Image1.jpg]

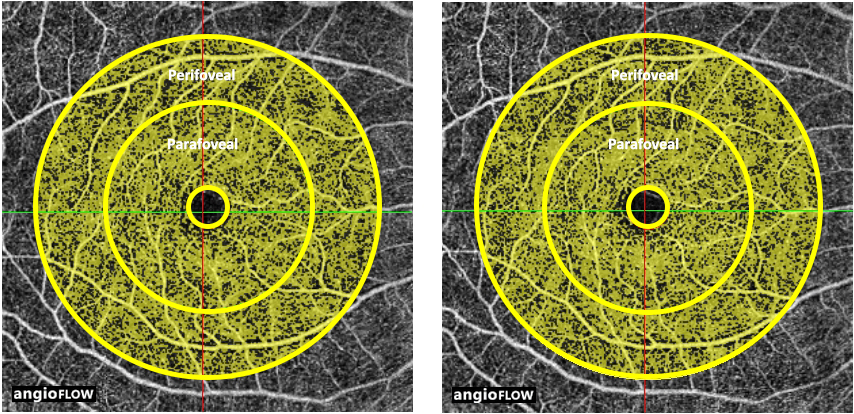

Supplement: Supplementary Figure 2 — Optic coherence tomographic angiogram of the macular area from a 29 year-old male; showing the perifoveal area, the annulus between the middle and outer circles, before (left, vessel density 71%) and after (right, vessel density 71%), and the parafoveal area, the annulus between the inner and middle circles, before (left, vessel density 80%) and after (right, vessel density 79%) the instillation of tropicamide eye drops. The yellow shading represents the perfused vessel density. [file Image2.JPEG]

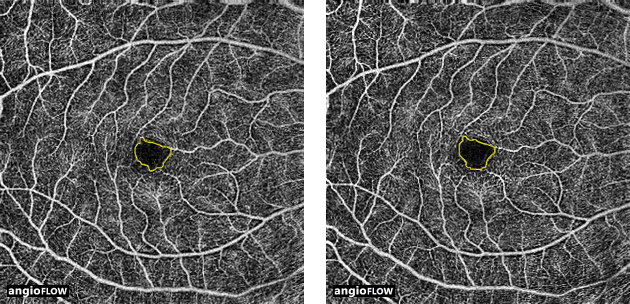

Supplement: Supplementary Figure 3 — Optic coherence tomographic angiogram of the foveal avascular zone (FAZ) from a 29 year-old male; showing outlined and measured FAZ before (left, 0.36 mm2) and after (right, 0.37 mm2) instillation of tropicamide/phenylephrine mixture eye drops. [file Image3.JPEG]
