# Supplementary material for: Phenylephrine Affects Peripapillary Retinal Vasculature—an Optic Coherence Tomography Angiography Study
Source: Front Physiol. 2017 Dec 4;8:996. doi: 10.3389/fphys.2017.00996 (PMC5722831; doi:10.3389/fphys.2017.00996)
Supplement: Supplementary file 4 [file Table1.DOCX]

**Supplementary Table 1. Systemic and ocular parameters**

**before and after instillation of tropicamide or tropicamide/phenylephrine mixture eye drops**

|  |  | Tropicamide | | | Tropicamide/Phenylephrine mixture | | |
| --- | --- | --- | --- | --- | --- | --- | --- |
|  |  | Pre-dilation | Post-dilation | P value | Pre-dilation | Post-dilation | P value |
| SBP (mmHg) | | 101.13 ± 17.63 | 106.63 ± 21.08 | 0.106 | 106.00 ± 13.62 | 106.63 ± 16.70 | 0.779 |
| DBP (mmHg) | | 72.50 ± 13.62 | 68.50 ± 13.58 | 0.172 | 67.63 ± 18.52 | 67.75 ± 16.83 | 0.916 |
| MAP (mmHg) | | 91.58 ± 16.04 | 93.92 ± 18.36 | 0.398 | 85.29 ± 15.90 | 79.29 ± 15.53 | 0.889 |
| HR (bpm) | | 80.88 ± 6.24 | 74.00 ± 10.89 | 0.093 | 74.75 ± 6.65 | 74.63 ± 5.29 | 0.866 |
| IOP (mmHg) | |  |  |  |  |  |  |
|  | Right (tested) eye | 13.11 ± 5.00 | 12.36 ± 4.83 | 0.237 | 12.75 ± 4.93 | 13.49 ± 3.28 | 0.624 |
|  | Left (control) eye | 13.33 ± 4.86 | 12.60 ± 4.19 | 0.207 | 13.08 ± 3.69 | 12.84 ± 2.56 | 0.779 |
| OPP (mmHg) | |  |  |  |  |  |  |
|  | Right (tested) eye | 78.47 ± 17.86 | 81.55 ± 18.32 | 0.263 | 80.46 ± 14.14 | 80.18 ± 16.33 | 0.944 |
|  | Left (control) eye | 78.26 ± 16.20 | 81.57 ± 17.74 | 0.263 | 80.13 ± 14.21 | 80.83 ± 16.80 | 0.889 |

Continuous variables are given as means ± SD. Wilcoxon matched-pairs signed-rank test was used to compare values obtained before and after administration of tropicamide or tropicamide/phenylephrine mixture eye drops.

SBP: systolic blood pressure; DBP: diastolic blood pressure; MAP: mean arterial pressure; HR: heart rate; IOP: intraocular pressure; OPP: ocular perfusion pressure
