# Supplementary material for: Phenylephrine Affects Peripapillary Retinal Vasculature—an Optic Coherence Tomography Angiography Study
Source: Front Physiol. 2017 Dec 4;8:996. doi: 10.3389/fphys.2017.00996 (PMC5722831; doi:10.3389/fphys.2017.00996)
Supplement: Supplementary file 5 [file Table2.DOCX]

**Supplementary Table 2. Individual data for retinal vessel density of the right eyes before and after the instillation of tropicamide/phenylephrine mixture eye drops**

| No | Gender | Age | Peripapillary | | | Perifovea | | | Parafovea | | | FAZ (mm^2^) | | |
| --- | --- | --- | --- | --- | --- | --- | --- | --- | --- | --- | --- | --- | --- | --- |
|  |  |  | Pre- | Post- | Change | Pre- | Post- | Change | Pre- | Post- | Change | Pre- | Post- | Change |
| 1 | Male | 26 | 85% | 85% | 0% | 71% | 80% | 9% | 74% | 83% | 9% | 0.35 | 0.36 | 0.01 |
| 2 | Female | 28 | 95% | 89% | -6% | 80% | 73% | -7% | 84% | 76% | -8% | 0.30 | 0.29 | -0.01 |
| 3 | Male | 29 | 90% | 78% | -12% | 79% | 73% | -6% | 81% | 76% | -5% | 0.38 | 0.38 | 0.00 |
| 4 | Female | 33 | 91% | 90% | -1% | 83% | 78% | -5% | 82% | 79% | -3% | 0.34 | 0.31 | -0.03 |
| 5 | Male | 36 | 82% | 83% | 1% | 71% | 71% | 0% | 71% | 72% | 1% | 0.36 | 0.39 | 0.03 |
| 6 | Male | 31 | 95% | 86% | -9% | 73% | 77% | 4% | 76% | 79% | 3% | 0.25 | 0.26 | 0.01 |
| 7 | Female | 24 | 88% | 81% | -7% | 72% | 66% | -6% | 74% | 72% | -2% | 0.18 | 0.18 | 0.00 |
| 8 | Female | 34 | 91% | 88% | -3% | 84% | 82% | -2% | 85% | 83% | -2% | 0.43 | 0.40 | -0.03 |

FAZ: foveal avascular zone.
